# Data on the morphology and chemical state of coatings based on TiN obtained by condensation with ion bombardment on various substrates

**DOI:** 10.1016/j.dib.2019.104737

**Published:** 2019-11-04

**Authors:** Petr M. Korusenko, Sergey N. Nesov, Sergey N. Povoroznyuk, Pavel V. Orlov, Dmitry N. Korotaev, Konstantin N. Poleschenko, Evgeny E. Tarasov

**Affiliations:** a Omsk State Technical University, Mira Avenue 11, 644050, Omsk, Russia; b Siberian State Automobile and Highway University, Mira Avenue 5, 644080, Omsk, Russia; c Federal Research and Production Center “Progress”, 5th Kordnaya Street 4, 644018, Omsk, Russia

**Keywords:** Condensation with ion bombardment, CIB, Titanium nitride, Coating, X-ray photoelectron spectroscopy, XPS, SEM

## Abstract

This article presents the effect of the substrate on the morphology and chemical composition of titanium nitride coatings formed using the condensation with ion bombardment method. Various steels, sintered hard alloy (tungsten carbide – 92%, cobalt – 8%) and titanium-based alloy were used as substrates. The paper presents the XPS data obtained at various depths from the surface. The article also presents the data of the wear resistance of coatings for road milling cutters.

**Table:** Specifications Table

Subject	Materials science Engineering
Specific subject area	Coatings. Formation of coatings by condensation with ion bombardment.
Type of data	Figure, Table
How data were acquired	SEM data and quantitative elemental EDX analysis of coatings on various substrates was carried out at the JEOL JSM 6610 LV scanning electron microscope. XPS data were registered at the Surface Science Center (RIBER) spectrometer at room temperature.
Data format	Raw, analyzed
Parameters for data collection	All coatings were formed at the HHB 6.6 equipment with the same application parameters (with the same time, cathode composition, residual medium and working gas composition).
Description of data collection	The changes in the morphology and chemical state of TiN coatings on various substrates by SEM, EDX and XPS were studied. XPS measurements were performed under ultrahigh vacuum conditions. SEM data were recorded at an accelerating voltage of 10 kV, with different spatial resolutions.
Data source location	Scientific and Educational Resource Center “Nanotechnology”, Omsk State Technical University, Omsk, Russia
Data accessibility	Data are available within this article

**Table:** 

**Value of the Data** • These data will expand the experimental base for studying the structural-phase composition of TiN coatings at different stages of their formation for various substrates. • The data could be used to study processes and develop technology for the formation of functional ion-plasma coatings by various physical vapor deposition methods (laser evaporation, thermal evaporation, magnetron sputtering, condensation with ion bombardment, etc.). • The data acquired can be used to select the parameters of TiN coating formation on the surface of products in order to increase wear resistance. • The data obtained will be useful in developing approaches to the formation of coatings based on TiN, because the type of substrate affects the thermal and diffusion processes during coating formation and the chemical composition of the substrate-coating interface. • Data on the chemical state of coatings at various depths from the surface can be used to analyze the processes of phase formation as well as diffusion of chemical elements in the process of coatings formation • The present data obtained can be used to modify a wide class of materials, because a dense coating is formed that adheres well to the substrate.

## 1 Data

The dataset of this article provides information on the effect of substrate on the morphology and chemical state of TiN coatings on steels, sintered hard alloy (WC – 92%, Co – 8%) and titanium-based alloy (Table 1). Also dataset provides information on elemental composition and chemical state as well as data of the wear resistance of coatings for road milling cutters.

**Table 1:** Coating on various substrates.

Sample name	Substrate (Russian classification)	Analog (Germany classification)
S1	Steel (110G13L)	GX120Mn12, GX120Mn13
S2	Sintered hard alloy (VK8)	HG30,HG40
S3	Steel (40X)	41Cr4
S4	Sintered hard alloy (VK8) with TiC coating	HG30,HG40
S5	Titanium-based alloy (VT-5)	3.7114, 3.7115

**Fig. 1:**
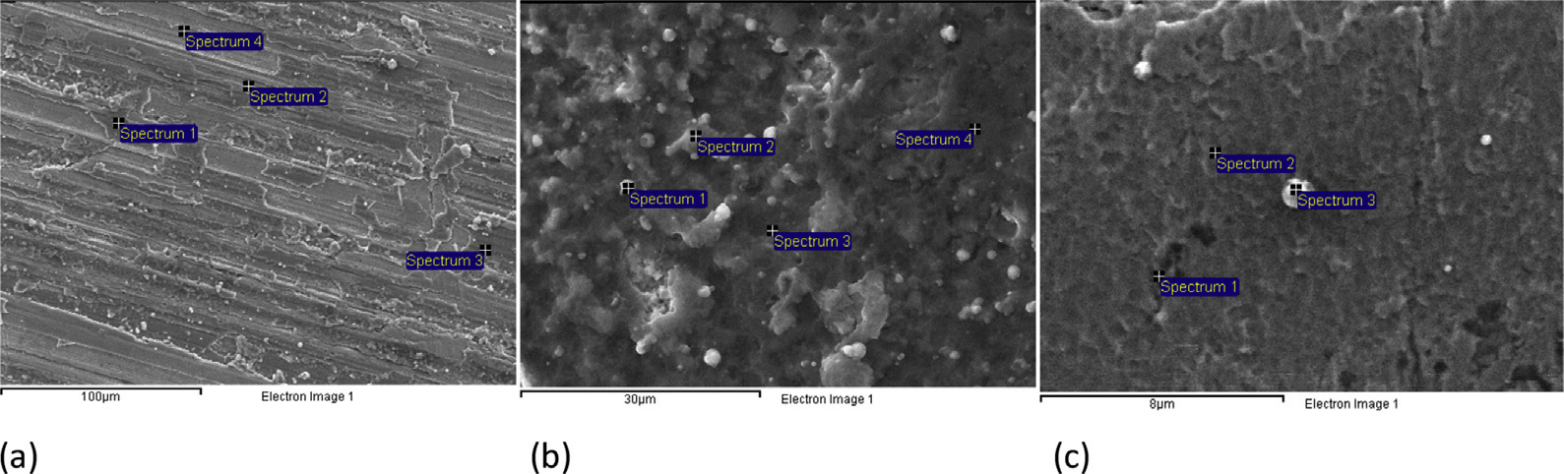
SEM images of the coatings obtained on various substrates: (a) – S1, (b) – S2, (c) – S3.

In Fig. 1 shows SEM images of the surface of S1–S3 samples. As can be seen (Fig. 1), the surface of the samples with coatings on various substrates has a different morphology. Elemental EDX analysis (Table 2) shows the presence of titanium, oxygen, nitrogen, carbon, and aluminum in the coatings composition. In addition, the presence of elements from substrates (Fe and Mn for S1; W and Co for S2; Fe, Cr, Si for S3) is observed. Due to the fact that the energies of x-ray quanta emitted from the K-levels of nitrogen and oxygen have close values, it is difficult to separately determine the values of their concentration. Therefore, in Table 2 shows the total values of the concentration of nitrogen and oxygen.

**Table 2:** Chemical composition of the coatings obtained on various substrates by EDX.

S1							
Spectrum	Concentration, at.%						
	[C]	[N] + [O]	[Al]	[Ti]	[Mn]	[Fe]	
1	12.19	39.54	0.68	39.56	1.54	6.49	
2	15.83	38.22	0.73	36.6	1.63	6.99	
3	12.23	42.02	0.71	37.67	1.38	5.99	
4	13.1	42.2	0.55	37.02	1.43	5.7	
S2							
Spectrum	[C]	[N] + [O]	[Al]	[Ti]	[Fe]	[Co]	[W]
1	16.86	44.49	1.52	31.51	0.05	0.48	5.09
2	15.54	48.92	0.76	28.18	0.07	1.53	5.00
3	19.50	46.80	0.62	25.27	0.05	2.65	5.11
4	18.99	46.70	0.56	23.63	0.13	7.27	2.73
S3							
Spectrum	[C]	[N] + [O]	[Si]	[Ti]	[Cr]	[Fe]	
1	5.75	23.32	1.65	15.94	1.98	51.36	
2	5.40	25.17	0.58	19.03	3.50	46.31	
3	8.08	22.66	0.39	23.07	3.56	42.24	

**Fig. 2:**
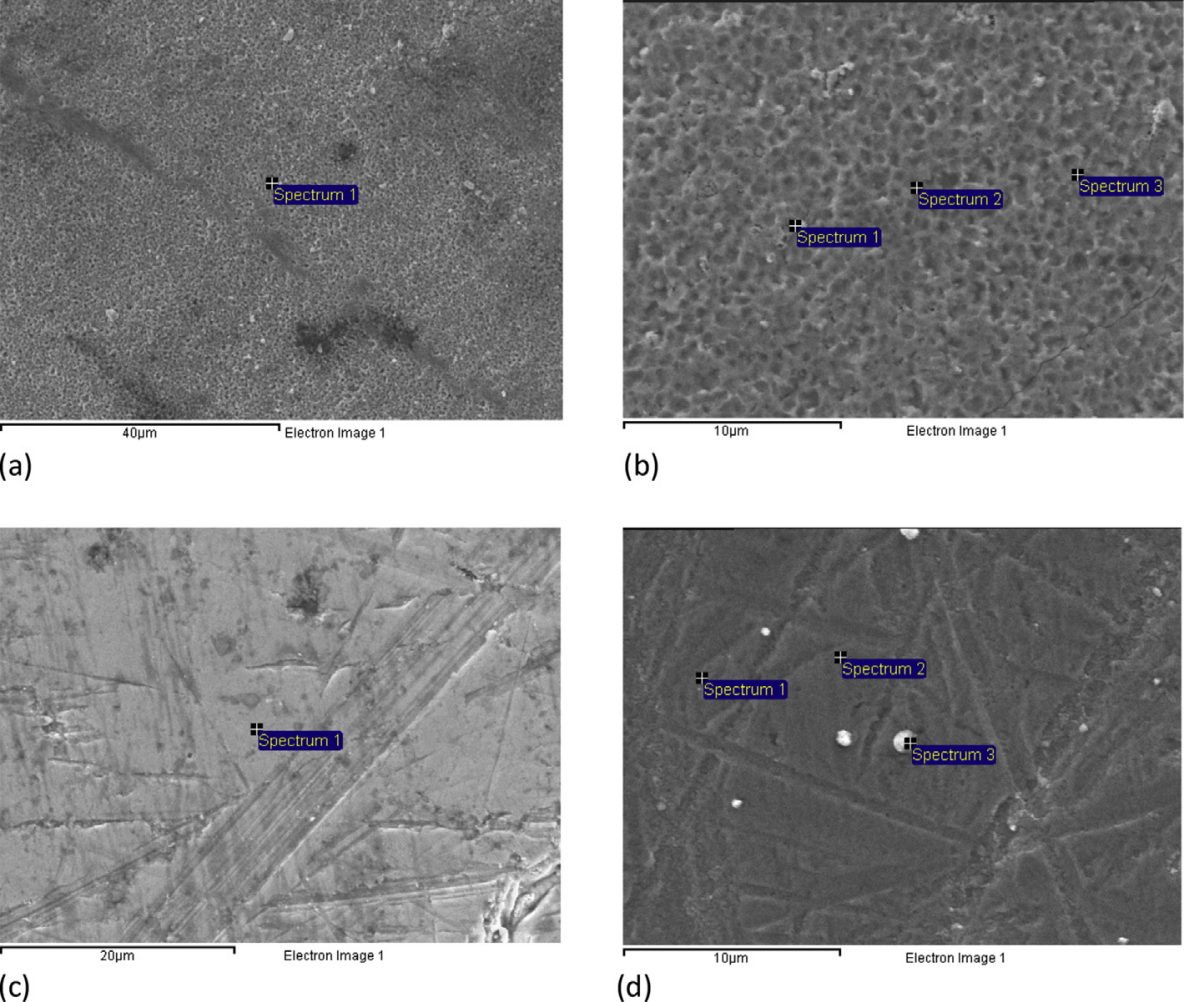
SEM images of the samples: (a) S4 without coating, (b) S4 with coating; (c) S5 without coating, (d) S5 with coating.

Fig. 2 shows SEM images of the surface of samples S4 and S5 before and after formation of coating. As can be seen, the coatings repeat the surface relief of the substrates. This indicates a high adhesion of coatings to substrates. In Table 3 shows the EDX data for samples S4 and S5.

**Table 3:** EDX data for the samples S4 and S5 before and after formation of coatings.

Sample S4 without coating				
Spectrum	[C]	[Ti]		
1	43.61	56.39		
S4				
Spectrum	[C]	[N+O]	[Ti]	
1	31.69	15.66	52.62	
2	32.12	12.56	55.25	
3	29.86	13.35	56.76	
Sample S5 without coating				
Spectrum	[C]	[O]	[Al]	[Ti]
1	0.82	7.51	9.23	82.44
S5				
Spectrum	[C]	[N+O]	[Al]	[Ti]
1	2.82	19.72	4.62	72.83
2	1.71	21.48	4.42	72.39
3	18.39	18.06	1.68	61.87

**Fig. 3:**
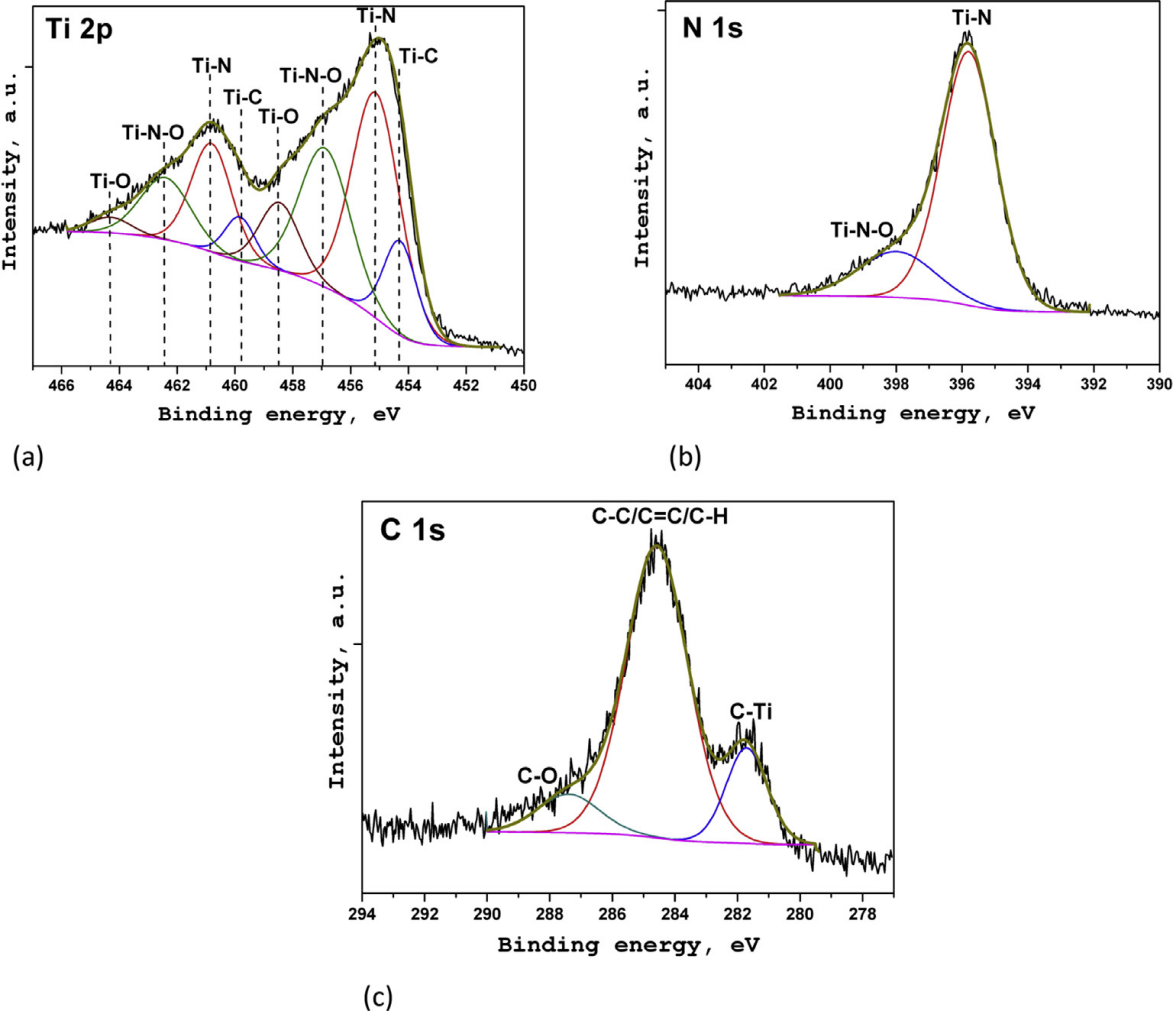
XPS spectra of Ti 2p (a), N 1s (b) and C 1s (c) for S2.

In the XPS survey spectra for each sample the lines corresponding to titanium, nitrogen, oxygen, and carbon are observed. The XPS spectra of the sample S2 were used for the detail chemical analysis of the coatings. On Fig. 3a the XPS spectrum of titanium (Ti 2p) is presented. The spectrum has two local maxima at binding energies of ∼455 eV (Ti 2p_3/2_) and v461 eV (Ti 2p_1/2_). According to data [1,2], the energy position of these maxima corresponds to titanium nitride. The intense components at the binding energies of ∼457 and ∼462 eV correspond to titanium oxynitride states [3,4]. The less intense components correspond to titanium oxide (maxima at the binding energies of ∼458 and ∼464 eV) [4], as well as titanium carbide (maxima at the binding energies of ∼454 and ∼460 eV), respectively [5].

The analysis of the N 1s XPS spectrum (Fig. 3b) shows the presence of two different chemical states of nitrogen in the coating. The most intense component at the binding energy of ∼396 eV corresponds to nitrogen in the titanium nitride [6]. The less intense component with a maximum at the binding energy of ∼398 eV is associated with the titanium oxynitride [6,7].

Fig. 3c shows the C 1s spectrum of carbon. The shape of the spectrum indicates the presence of carbon in the three chemically nonequivalent states. The high-intensity component of the spectrum at the binding energy of ∼284.6 is corresponds to the carbon in С-С (sp^3^), С = C (sp^2^) and C–H chemical bonds [8]. The component with a maximum at the binding energy of ∼287.4 eV corresponds to the carbon in chemically bonds to oxygen [9]. A spectrum component with a maximum at the binding energy of ∼281.7 eV corresponds to the carbon chemically bonds to titanium (titanium carbide) [10].

**Fig. 4:**
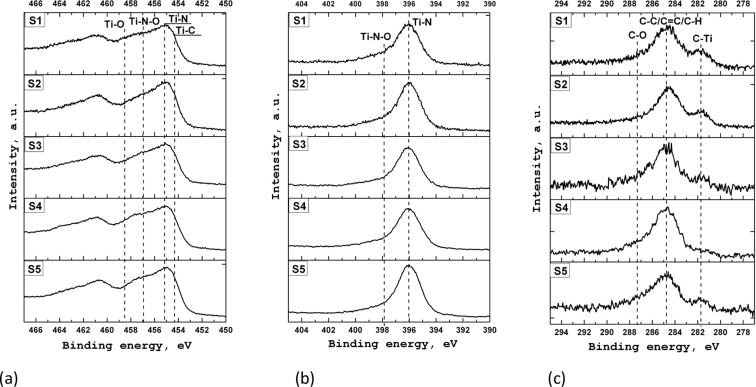
XPS spectra of titanium (a), nitrogen (b) and carbon (c) for S1–S5.

Fig. 4 shows the XPS spectra of titanium, nitrogen, and carbon for the samples S1–S5. An analysis of the XPS spectra indicates that the chemical state of the elements in the coatings obtained on various substrates is fairly close. However, in the case of the samples S1, S4, and S5, in the Ti 2p_3/2_ spectrum (Fig. 4a) in the range of binding energies of 457–459 eV a higher intensity is observed. Moreover, the shape of the nitrogen line for all samples is identical (Fig. 4b). This indicates that coatings on the samples S1, S4, and S5 contain more titanium oxides than coatings on the samples S2 and S3. Analysis of C 1s lines (Fig. 4c) showed that the content of titanium carbides in the coatings is slightly different. The highest intensity of C–Ti states is observed for sample S1.

**Fig. 5:**
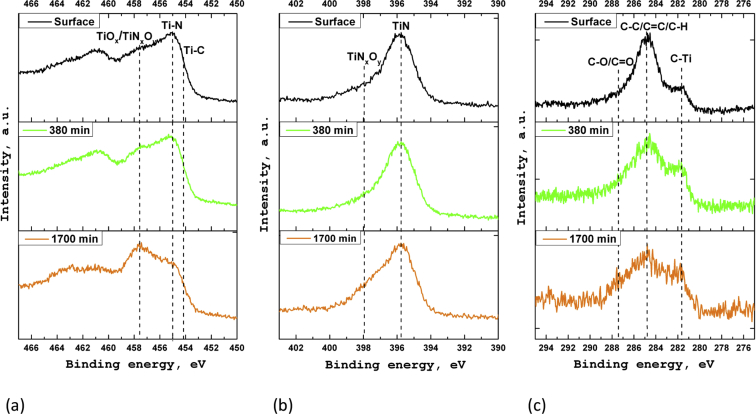
XPS spectra of titanium (a), nitrogen (b) and carbon (c) for S1 at various ion sputtering time.

Fig. 5 shows the XPS spectra of Ti 2p, N 1s, and C 1s, for sample S1, measured after ion sputtering. An analysis of these spectra indicates the heterogeneity of the coatings composition in depth. As can be seen, with an increase in the sputtering time, an increase in the relative part of oxynitride and titanium carbide in the coating composition is observed.

**Fig. 6:**
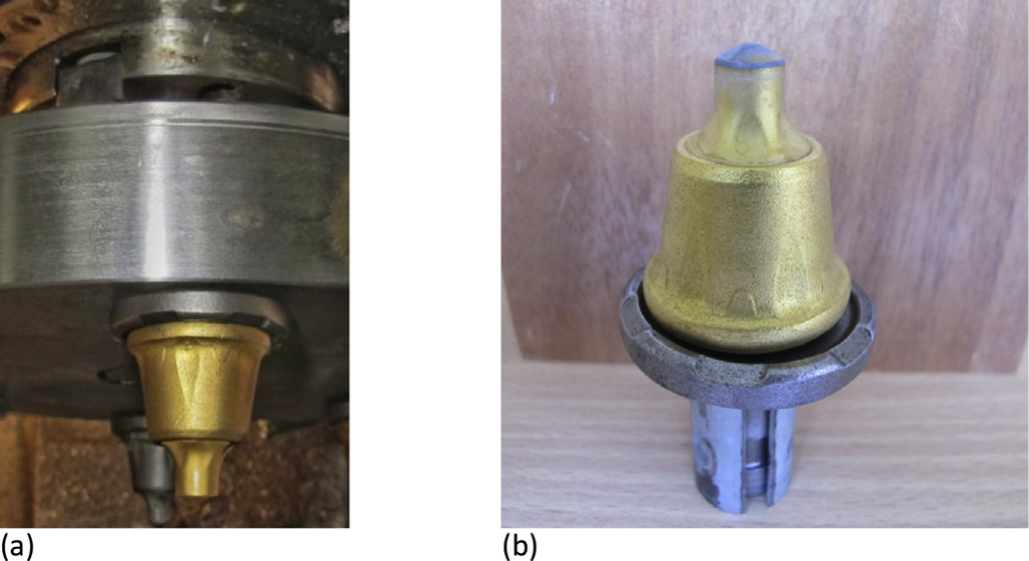
Images of the road mills cutters before (a) and after tests (b) of wear resistance.

**Fig. 7:**
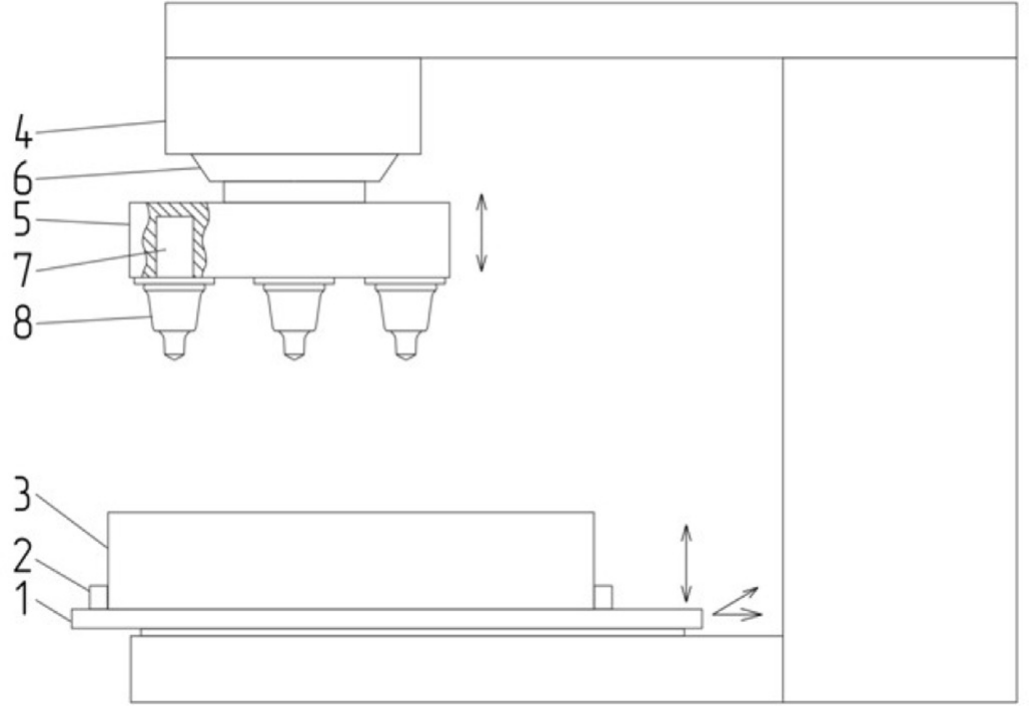
Scheme of the test bench for wear resistance: (1) – movable table with the ability to move in a horizontal and vertical plane; (2) – mount of a sample; (3) – sample of asphalt concrete pavement; (4) – driving motor; (5) – cutter holders; (6) – landing cone; (7) – landing slots for cutters; (8) – cutters.

Fig. 6 shows the images of the road mills cutters before and after the tests of wear resistance. These cutters are used to remove a layer of deformed asphalt concrete during overhaul and maintenance of roads. The cutters are made of sintered hard alloy (corresponds to sample S2) with the TiN-based coating formed by the CIB method. Tests of the samples for wear resistance were carried out on the special bench simulating the work of road milling machines (Fig. 7) when processing concrete with an average compressive strength of 4490 N/cm^2^. Test data showed that coated cutters provided an increase in their wear resistance by 20%.

## 2 Experimental design, materials, and methods

In this work, the following substrates were used: steels (110G13L and 40X), sintered hard alloy (VK8) without and with TiC coating as well as titanium-based alloy (VT-5) wafer. The formation of titanium nitride coatings on various substrates, the HHB 6.6 equipment was used. The cathode of metallic titanium with the inclusion of metallic aluminum was used, the presence of which reduces the likelihood of the formation of a droplet phase of metallic titanium in the coating [11]. Prior to coating, the substrates were preliminarily ion-sputtered by applying a high voltage of ∼900–1000 V at a chamber pressure of ∼5·10^−5^ Torr. Moreover, in the process of preliminary cleaning, the samples were heated to temperatures of ∼450–550°С and their surface was activated due to the formation of structural defects. The coatings were formed in an atmosphere of dry nitrogen at a pressure in the chamber of ∼2–3·10^−3^ Torr. The arc discharge current was ∼100–110 A, and the voltage on the substrate was ∼200–220 V. The coatings formation time was 15 minutes.

An analysis of the morphology and structure of the coatings was performed using the SEM method on the JEOL JSM 6610 LV electron microscope at the Omsk Regional Shared Equipment Center SB RAS. The SEM images were recorded at the accelerating voltage of 10 kV, with different spatial resolutions. EDX analysis was performed on the JEOL JSM 6610 LV microscope using INCA-350 Oxford Instruments. The diameter of the electron beam in the EDX study was ∼1.5 μm, and the analysis depth to ∼2–3 μm.

The composition and chemical state of the coatings were studied using the XPS method at the Surface Science Center (Riber) analytical complex. To excite X-ray radiation, an AlKα source with the energy of 1486.6 eV was used. XPS spectra were obtained under ultrahigh vacuum (∼10^−9^ Torr) using the MAC-2 two-stage cylindrical mirror analyzer. The diameter of the x-ray beam was about 5 mm, and the source power was 240 W. The depth of analysis was ∼1–3 nm. To obtain information on the composition and chemical state of the coating elements in the near-surface region of the samples, a layer-by-layer XPS analysis was used, which was carried out directly in the spectrometer chamber. The coating layer was sputtering by the argon ion beam with the average energy of 3 keV at the pressure in the spectrometer chamber of ∼3·10^-5^ Torr. The sputtering rate of the coatings was ∼ 1–2 nm/min.

Testing for wear resistance of cutters based on sintered hard alloy (VK8) coated with titanium nitride was performed on the special bench, which simulating the work of road milling machines, when processing concrete with an average compressive strength of 4490 N/cm^2^. The rotational speed of the milling cutter was 200 rpm. The working width of the processed material was 10 mm. The cutting depth was 4 mm, and the feed was 80 mm/min.
